# Plant-mediated effects of ozone on herbivores depend on exposure duration and temperature

**DOI:** 10.1038/s41598-019-56234-z

**Published:** 2019-12-27

**Authors:** Laura Duque, Erik H. Poelman, Ingolf Steffan-Dewenter

**Affiliations:** 1 Department of Animal Ecology and Tropical Biology, Biocenter, University of Würzburg, Würzburg, Germany; 2 Laboratory of Entomology, Wageningen University, Wageningen, The Netherlands

**Keywords:** Ecology, Environmental impact

## Abstract

Abiotic stress by elevated tropospheric ozone and temperature can alter plants’ metabolism, growth, and nutritional value and modify the life cycle of their herbivores. We investigated how the duration of exposure of *Sinapis arvensis* plants to high ozone and temperature levels affect the life cycle of the large cabbage white, *Pieris brassicae*. Plants were exposed to ozone-clean (control) or ozone-enriched conditions (120 ppb) for either 1 or 5 days and were afterwards kept in a greenhouse with variable temperature conditions. When given the choice, *P. brassicae* butterflies laid 49% fewer eggs on ozone-exposed than on control plants when the exposure lasted for 5 days, but showed no preference when exposure lasted for 1 day. The caterpillars took longer to hatch on ozone-exposed plants and at lower ambient temperatures. The ozone treatment had a positive effect on the survival of the eggs. Ozone decreased the growth of caterpillars reared at higher temperatures on plants exposed for 5 days, but not on plants exposed for 1 day. Overall, longer exposure of the plants to ozone and higher temperatures affected the life cycle of the herbivore more strongly. With global warming, the indirect impacts of ozone on herbivores are likely to become more common.

## Introduction

Ozone is a highly oxidative gas with widely recognized detrimental effects on human health^1^ and plant growth^2^. Far less documented are the effects of ozone on plant-animal interactions (but see^3–14^ for some examples).

Tropospheric ozone is a secondary gaseous pollutant that forms through photochemical reactions of its precursors (nitrogen oxides and volatile organic compounds)^8^. Despite the efforts to reduce precursor emissions in some parts of the world, there is a high probability that, globally, the average concentration of tropospheric ozone is still going to increase in the coming years^15^. Rising precursor emissions in rapidly growing economies, mainly in Asia, and global warming are expected to be the source of the problem^15^. Also, controlling precursor emissions locally is not as effective in reducing ozone concentrations as desired due to long-range transport of pollution^16^. Furthermore, simulations for the year 2050 point to increased frequency of high ozone episodes in developing regions in the best-case scenario, but to a generalized increase in ozone episodes when the use of fossil fuels keeps increasing^17^.

Tropospheric ozone is reported to affect plant photosynthesis, with consequences for plant growth, plants’ nutritional value and crop yield^18^. Many cases of visible plant injury and biomass reduction due to ozone exposure have been reported in Europe, with 39% of these happening in crops^19^. The estimated global economic losses deriving from yield losses of wheat, soybean and maize for the year 2000 ranged between $11 and 18 billion^20^ and are expected to increase by $1 to 17 billion in 2030, depending on the metrics used and the emission scenarios considered^21^.

By changing the plants’ metabolism, ozone may indirectly affect the interaction between plants and other organisms through quantitative or qualitative changes in the production of plant secondary metabolites involved in the communication between plants or in plant attraction/repellence/defence^4,6,22^. Ozone may also react with volatile secondary metabolites in the atmosphere, breaking them down into unknown reaction products, possibly disrupting the communication with other organisms^7,23–25^. Furthermore, ozone may change the nutritional value or the toxicity of the plants, altering the performance of herbivores feeding on these plants^5,9,10,12,26,27^.

In this study, we focus on the interaction between the herbivore *Pieris brassicae* L. (large cabbage white) and the host plant *Sinapis arvensis* L. (wild mustard). *P. brassicae* is a butterfly whose caterpillars are specialist herbivores feeding on Brassicaceae plants that contain glucosinolates^28^. Wild mustard, *S. arvensis* is one of those plants. It is indigenous to Europe, the Middle East and Western Asia and is nowadays an important weed of field crops in most of the temperate regions of the world^29^.

The interaction between *S. arvensis* and *P. brassicae* starts at the point when a female butterfly searches for and chooses a suitable place for laying her eggs. This process is usually guided by chemical cues produced by the plants^30^. Once a choice is made, the butterfly usually lays its eggs in clusters attached to the under surface of leaves^31^. After hatching, the insects use the plant as food source. From the plants perspective, the larval stage of the herbivore is an antagonist, and may induce a defensive response, both at egg and caterpillar stage. Plant defence mechanisms can be induced by egg deposition or caterpillar damage and include a hypersensitive response-like necrosis that kills the eggs by desiccation (direct defence), the production of toxins (direct defence) or the production of chemical cues for recruiting parasitoids (indirect defence)^32–34^.

Plant stressors, such as ozone, may affect each of these interactions. Studies on the effects of ozone on the oviposition preference of insects revealed that exposure of host plants to ozone had positive^35^, negative^6,36,37^ or no effects^11^ on the oviposition preference of the insects. Furthermore, one study showed that ozone has an effect on the fecundity of the females^9^. Also, several studies have reported a plant-mediated effect of ozone on caterpillar development, be it a positive^27,38^ or a negative one^5,39^. In some cases, previous exposure of the plant to ozone altered the consumption of plant material^5,36^.

Factors that may explain contradicting findings are the ozone exposure level and interactive effects with temperature. Ozone exposure is defined by the concentration of ozone, the frequency and the duration of exposure. Previous studies on the effects of ozone on plant-insect interactions considered acute exposure^5,36^ (for a short period but with very high ozone concentrations) or chronic exposure^3,9–11,14^ (often covering most of the life span of the plant, with concentrations that usually do not exceed 2 times the ambient ozone concentrations). However, the duration of exposure is not considered as a factor in these studies. Therefore, it is uncertain, particularly for acute exposures, whether the effects of ozone would be additive or there would be recovery and a compensatory mechanism after an initial response.

Furthermore, little is known about the interactive effects of elevated ozone and temperature^40^. Although elevated temperature increases plant growth, the combined effects of temperature and ozone on plants seem to be case-specific^41^. Elevated temperature also increases insect metabolism^42^, affecting plant-insect interactions through changes in consumption^43^. Studies that integrate the effects of both temperature and ozone on plant-insect interactions are missing, although higher mean temperatures under future climatic conditions may reinforce or attenuate the effects of ozone on these interactions.

In this study we performed a greenhouse experiment to assess the response of *Pieris brassicae* to *Sinapis arvensis* plants exposed to 2 levels of ozone (0 and 120 ppb) for 2 different periods (1 and 5 days). Specifically, we address the following questions: (1) Does a previous exposure of plants to ozone affect: (a) the oviposition preference of the butterfly?; (b) the duration of the egg stage?; (c) the egg survival rate?; (d) the caterpillar performance? (2) Does the duration of exposure influence the way ozone affects these parameters of the herbivore life cycle? (3) How is temperature modulating the way ozone affects these parameters?

## Material and Methods

### Biological material

Wild-type *Sinapis arvensis* seeds were provided by the Botanical Garden of Konstanz, Germany. Wild mustard plants were grown in 18 × 18 cm pots in a 2:1-mixture of peat based substrate (Einheits Erde CL ED 73) and sand (Hamann Filtersand 0,7–1,25 mm). The plants grew in a greenhouse in the Biocenter of the University of Würzburg under 50%/80% relative humidity and 16 h/8 h, light/dark respectively. To keep the photoperiod constant, supplementary illumination was applied by high-pressure sodium lamps whenever the light intensity outside the greenhouse would drop below 20 kLux. Although this greenhouse can be heated to increase the temperature, the aeration system does not allow to decrease the temperature in warm, sunny days. During this experiment, the temperature was recorded every 12 minutes and later included as a potential explanatory variable. The overall temperatures in the greenhouse ranged between 14.0 and 35.1 °C.

*Pieris brassicae* butterflies were obtained from a population that is routinely reared on *Brassica oleracea* var. *gemmifera* cultivar Cyrus in the Laboratory of Entomology at Wageningen University, The Netherlands. As the experiment continued, we also used butterflies reared in our greenhouse, descendants from the same population. The butterflies were kept in the greenhouse, inside a 1.15 m-high insect rearing tent (Bugdorm) and were fed a 10–20% honey solution.

### Ozone exposure system

**Figure 1:**
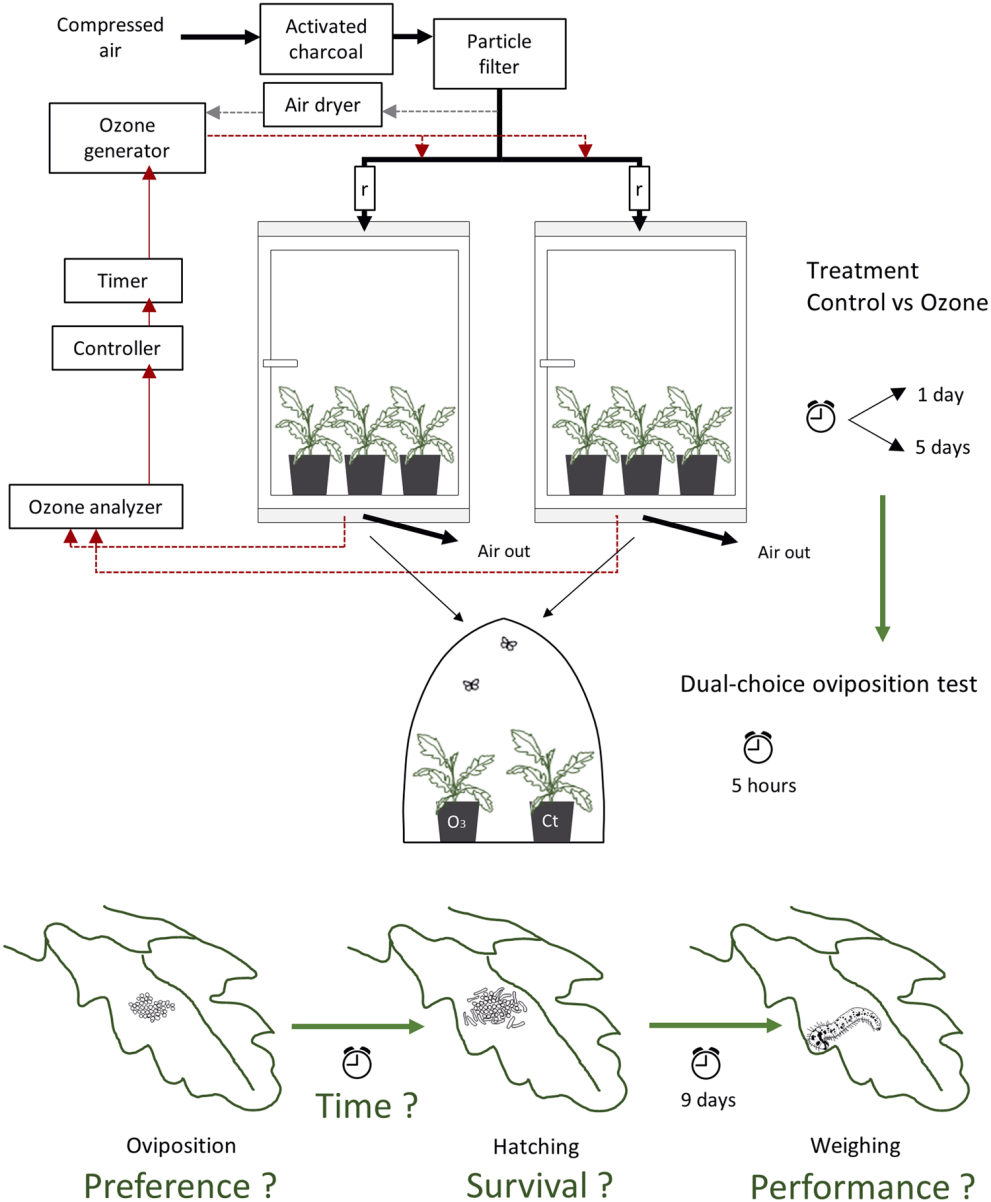
Representation of the ozone-exposure system, the experiment and the main research questions. r - rotameter.

In the greenhouse, we installed an ozone-exposure system (Fig. 1) consisting of: (1) 2 glass chambers with a stainless steel door frame and an approximate volume of 1000 L each; (2) a customized ozone generator (INNOTEC high engineering GmbH) linked to an air dryer (AIRdryer3.2, INNOTEC); (3) an ozone analyser (APOA-370, Horiba Ltd.); (4) a controller that links the ozone analyser and the ozone generator, allowing us to regulate the ozone concentration in the chamber; (5) a timer; (6) a main air stream of compressed air passing through an activated charcoal filter and a particle filter before it reaches the chambers; (7) a secondary air stream, branching from the main one, passes through the air dryer and the ozone generator and disembogues in the main air stream, allowing for ozone enrichment of the incoming air; (8) 2 rotameters allow us to level the amount of incoming air to each chamber to 70 L/min. Because the chambers are almost exclusively made of glass, the photoperiod conditions are the same as in the greenhouse itself.

The two chambers allow to have, at any given time, an ozone-clean environment and an ozone-enriched environment, and the treatments are interchangeable. The ozone analyser is constantly sucking in the air from one of the chambers, analysing it for its ozone content. This information is then passed on to the controller that gives feedback to the ozone generator, switching it on or off in order to achieve the desired average ozone concentration, which can be modified in the controller. Pre-experiment tests revealed that the ozone concentration of the clean air chamber ranges between 0 and 1 ppb. Therefore, the ozone concentration is only monitored in the ozone-enriched chamber.

### Study design

This study was performed between February and May 2018. The plants were fumigated before being presented to the herbivores. The fumigation treatments consisted of exposing the plants to 2 levels of ozone (control vs ozone) for 2 different periods (1 or 5 days). In the control treatment plants were exposed to clean air (~0 ppb ozone) for the entire day, whilst in the ozone treatment, the plants were exposed to elevated-ozone conditions (~120 ppb ozone, Supplementary Table S1) for 6 hours/day, between 11 h 00 and 17 h 00, and to clean air for the rest of the day. 240 µg.m^−3^ (~120 ppb) is the alert threshold for ozone established by the European Union. In Europe, exceedances of this value occur usually in summer and, although uncommon, several days with exceedances may be observed in the period of one year^44–46^.

At the start of each experimental round, the duration of the exposure was fixed and each fumigation chamber was randomly assigned a treatment (control vs ozone). Four weeks after sowing, eight plants were placed in each chamber and exposed to the corresponding treatment.

Following ozone exposure, one plant from each treatment was enclosed in an insect-rearing tent along with one female and one male *P. brassicae* butterfly. The butterflies remained in the tents for 5 hours, after which the number of eggs laid on each of the two plants were counted and the plants were measured. The health status of the plant was recorded: percentage of leaves showing signals of visible ozone damage (chlorosis/necrosis) and percentage of leaves infected by powdery mildew, a fungal infection. Each butterfly was only used for one oviposition test. Plant pairs that were offered to butterflies that did not lay any eggs during the 5-hour oviposition period were excluded from the oviposition preference analysis.

After the oviposition preference tests, all plants were kept in a greenhouse compartment on plant saucers surrounded by water to avoid hatched caterpillars to migrate to a different plant. The plants were monitored daily for detection of caterpillar hatching.

The duration of the egg phase was calculated for the eggs laid on each plant, as the number of days between the day of the oviposition tests and the day when the majority of the caterpillars hatched. When none of the eggs laid by a butterfly hatched, we considered that it was likely that they were not fertile and they were excluded from the analysis of egg survival rate. This was the case for 4 control and 3 ozone replicates for 1-day exposed plants and 2 control and 2 ozone replicates for 5-day exposed plants.

After hatching, all caterpillars were retrieved from the plants and counted. Afterwards, 10 caterpillars were reintroduced on the plants where they had hatched. Plants that had no eggs or that had eggs that did not hatch also received 10 caterpillars from plants of the same treatment. In both cases the newly hatched caterpillars were introduced on the fourth oldest leaf. Nine days after the introduction of the caterpillars, they were removed from the plants and weighed.

In total, we performed 13 experimental rounds: 6 for the 1-day treatment and 7 for the 5-day treatment. One of the 1-day treatment rounds was not completed since the caterpillars did not hatch, presumably due to desiccation and, therefore, we had no larvae to reintroduce.

### Statistical analysis

All statistical analyses were performed using R^47^ (version 3.4.2). The data collected for the 1-day and 5-day exposed plants were analysed separately, with test results and model fitting presented separately for each duration of treatment.

A binomial test to compare proportions of injured plants was performed to evaluate the effect of the ozone treatment on plant injury (prop.test function).

We used the dredge function (MuMIn package^48^, version 1.42.1) to assess which models best explain our data. The equations in this section show the variables that were considered as potential predictors in the global models.

For the oviposition preference tests, we used the number of eggs laid on the plants as the response variable. We fitted a generalized mixed effects model for zero-inflated data with butterfly as the random factor (glmmTMB, glmmTMB package^49^, version 0.2.2.0, family = negative binomial).

$$Number\,of\,eggs\,laid \sim Treatment\,\ast \,fungal\,infection+Plant\,height$$

To check if the tendency observed on the number of eggs laid per plant was kept after hatching, we refitted the generalized mixed effects model with zero-inflated data and butterfly as the random factor for the number of caterpillars hatched per plant (glmmTMB, family = negative binomial).

$$Number\,of\,caterpillars\,per\,plant \sim Treatment\,\ast \,fungal\,infection$$

We used a generalized mixed effects model with butterfly as a random factor to analyse the egg survival rate (glmer, lme4 package^50^, family = binomial, link = logit).

$$\begin{array}{c}Egg\,survival \sim Treatment\,\ast \,Average\,temperature\,during\,the\,egg\,stage\\ \,+Treatment\,\ast \,Number\,of\,eggs\,per\,plant\end{array}$$

The duration of the egg stage was analysed, by fitting a linear mixed effects model with butterfly as the random factor (lmer, lme4 package^50^, version 1.1–17).

$$\begin{array}{c}Duration\,of\,egg\,stage \sim Treatment\,\ast \,Average\,temperature\,during\,the\,egg\,stage\\ \,+Treatment\,\ast \,Number\,of\,eggs\,per\,plant\end{array}$$

Fungal infection was not included in the global models to analyse the duration of the egg stage and the egg survival because infection rate was highly correlated with the average temperature during the egg stage and the latter revealed a stronger predictor for both variables than fungal infection. We took butterfly as a random factor in the previous models, to account for (1) the dependency in the number of eggs laid on the two plants exposed to the same butterfly, (2) the fact that eggs laid by the same butterfly are expected to have similar quality, leading to similar egg survival and similar duration of the egg stage.

The weight of the caterpillars was cube root-transformed in order to approximate a normal distribution and was analysed with a linear mixed effect model (lmer) with the plant as random factor.

$$\begin{array}{c}Caterpillar\,weight\sim Treatment\,\ast \,Number\,of\,eggs\,per\,plant\\ \,+Treatment\,\ast Average\,temperature\,during\,the\,caterpillar\,stage\\ \,+Treatment\,\ast \,fungal\,infection\end{array}$$

The number of eggs was included as a potential predictor for caterpillar weight, because egg deposition was shown to affect the development of caterpillars in several species of Brassicaceae^51^.

Model validation was performed by visually checking the residuals for the linear models and by simulating residuals with the Dharma package^52^ (version 0.2.0) for the generalized linear models.

In the results’ section we present the models with the lowest AICc for each research question. Alternative models (ΔAICc < 2.0) are shown in the supplementary material (Supplementary Tables S2–S6). The number of replicates for each research question is provided in Table S7.

## Results

We observed signs of plant injury on plants exposed to ozone. While 2% of the plants exposed to ozone for 1 day and 39% of the plants exposed to ozone for 5 days showed chlorosis/necrosis in several degrees, none of the control plants showed visible injury. This indicates a strong effect of ozone on plant injury for a 5-day long treatment (χ^2^ = 24.945, df = 1, p < 0.001), but not for a 1-day long treatment.

**Figure 2:**
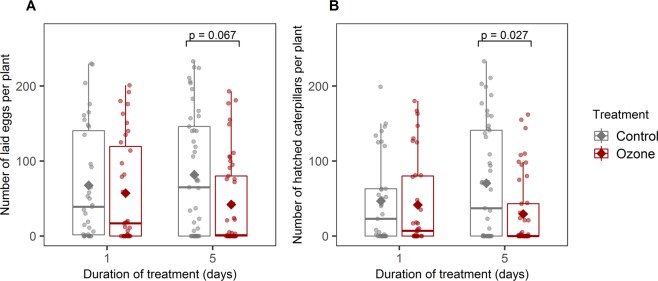
Plant-mediated effects of 2 levels of ozone exposure (1 and 5 days) on (**A**) the number of eggs laid by *Pieris brassicae* butterflies on dual-choice assays and (**B**) the number of caterpillars per plant after hatching. The dots represent the data points, the diamonds correspond to the means. P-values for the effect of the treatment, given by the reported models, are shown.

During the 5-hour periods of the oviposition dual-choice assays, each female butterfly laid between 1 and 233 eggs, with a mean of 96 eggs. When given the choice between a plant previously exposed to ozone and a control plant, butterflies chose to either lay all the eggs on one of the plants or distribute them between the two plants they were offered. The butterflies laid 15% fewer eggs on plants exposed to ozone for 1 day and 49% fewer eggs on plants exposed to ozone for 5 days than on the respective control plants (Fig. 2A). The best model based on AICc (Table 1) indicates an effect of ozone on the oviposition preference of the butterfly when the plants were exposed for 5 days (z = −1.830, p = 0.067), but not when the plants were exposed for only one day (Fig. 2A, Table 1). The health status of the plant also affected the number of eggs laid, with fewer eggs being laid on plants with a higher fungal infection rate (Table 1, z = −2.346, p = 0.019 and z = −2.088, p = 0.037, for 1- and 5-day exposed plants respectively).

**Table 1:** Summary of the models with the lowest AICc for each response variable.

Predictors	Response variable									
	Number of laid eggs		Number of caterpillars on plants		Egg survival		Duration of egg phase		Larval weight	
**1 day**	**z**	**P**	**z**	**P**	**z**	**P**	**t**	**P**	**t**	**P**
Treatment	—	—	—	—	2.962	**0.003**	2.127	**0.040**	—	—
Fungal infection	−2.346	**0.019**	−2.248	**0.025**					—	—
Plant height	—	—								
Temp egg					1.923	**0.055**	−17.27	**<0.001**		
Temp cat									22.950	**<0.001**
Number of eggs					1.885	**0.059**			—	—
Treat x Fung inf	—	—	—	—					—	—
Treat x temp egg					−2.796	**0.005**	—	—		
Treat x temp cat									—	—
Treat x nr eggs					—	—	—	—	—	—
**R**^2^					0.62		0.88		0.89	
**5 days**	**z**	**P**	**z**	**P**	**z**	**P**	**t**	**P**	**t**	**P**
Treatment	−1.830	**0.067**	−2.209	**0.027**	2.352	**0.019**	2.017	**0.062**	2.76	**0.007**
Fungal infection	−2.088	**0.037**	—	—					−3.141	**0.002**
Plant height	—	—								
Temp egg					—	—	−4.157	**<0.001**		
Temp cat									12.102	**<0.001**
Number of eggs					2.633	**0.008**			—	—
Treat x Fung inf	—	—	—	—					—	—
Treat x temp egg					—	—	—	—		
Treat x temp cat									−2.856	**0.005**
Treat x nr eggs					—	—	—	—	—	—
**R**^2^					0.74		0.90		0.88	

Treat = Treatment (Control vs Ozone); Fung inf = Fungal infection (percentage of leaves infected by powdery mildew); Temp egg = average ambient temperature during the egg stage; Temp cat = average ambient temperature during the caterpillar stage. —Indicates variables that were considered for the best model but were not included in the model with the lowest AICc. Empty cells indicate variables that were not considered for the best model. We present t or z-values, depending if the fitted model is a linear mixed effects model or a generalized linear mixed effects model, respectively.

After hatching, the number of caterpillars was 11% and 58% lower on ozone-exposed plants than on control plants, for 1- and 5-day exposed plants respectively. However, the effect of ozone was only significant for the longer exposure (z = −2.209, p = 0.027; Fig. 2B).

**Figure 3:**
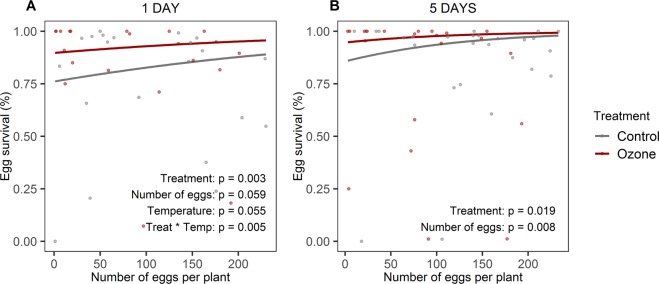
The effect of ozone and the number of eggs per plant on the survival rate of the eggs deposited on (**A**) 1-day exposed plants and (**B**) 5-days exposed plants. The lines represent the reported models’ regression lines and the dots are the data points. The p-values for each predictor in the reported models are shown.

The ozone treatment had a positive effect on the survival of the eggs (z = 2.962, p = 0.003 and z = 2.352, p = 0.019, for 1- and 5-day exposed plants respectively, Fig. 3A,B). The survival was also positively affected by the number of eggs laid per plant (z = 1.885, p = 0.059 and z = 2.633, p = 0.008, for 1- and 5-day exposed plants respectively, Fig. 3A,B). The ambient temperature during the egg stage had a positive effect on the survival of eggs laid on 1-day exposed plants (z = 1.923, p = 0.055). A significant interaction between the treatment and the temperature during the egg stage (z = −2.796, p = 0.005), revealed that the temperature only affected the survival of the eggs laid on control plants (Supplementary Fig. S1).

**Figure 4:**
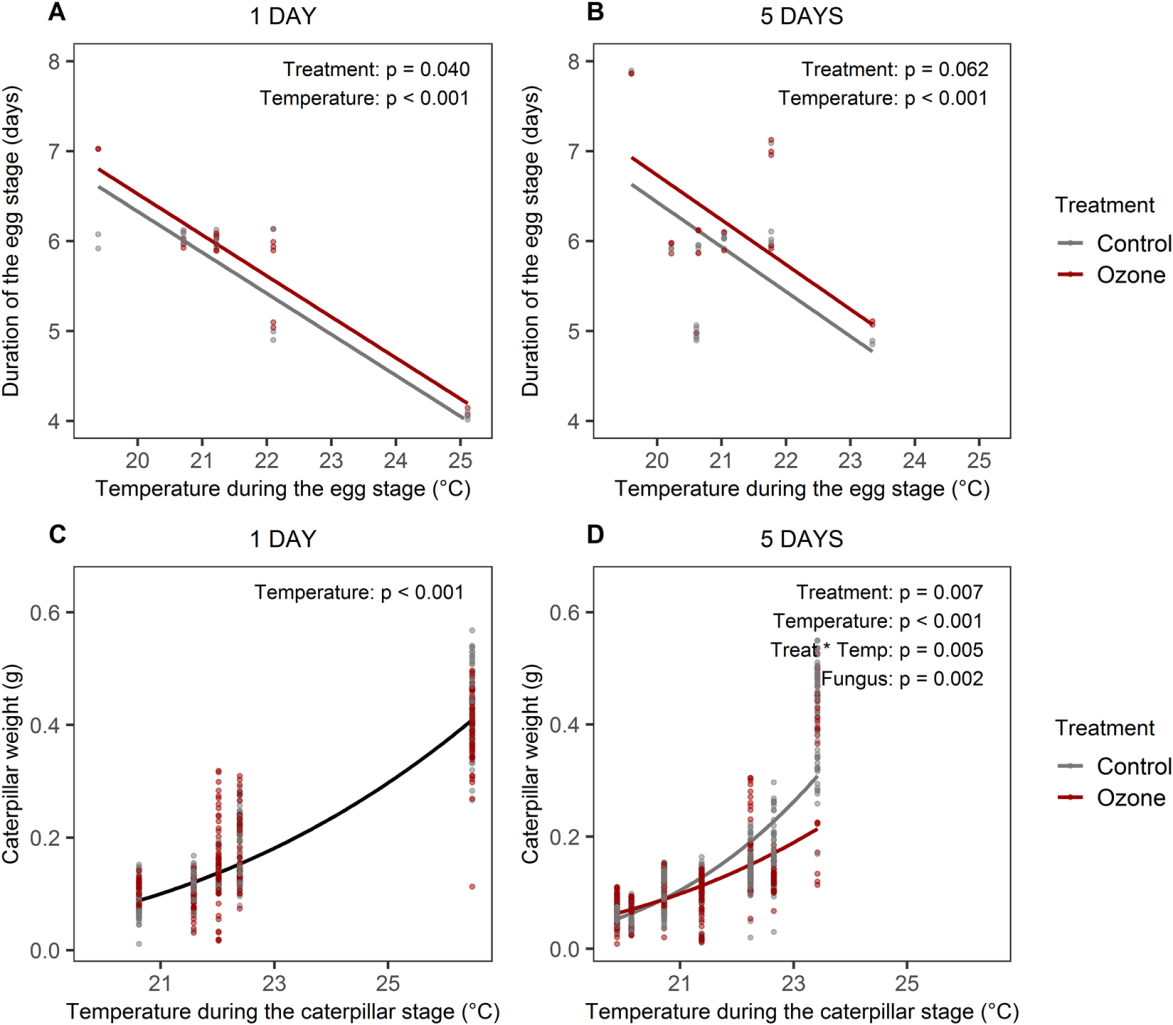
The effect of ozone and temperature on the duration of the egg stage (**A**,**B**) and the caterpillar weight (**C**,**D**). (**A**,**C)** Refer to 1-day exposed plants and (**B**,**D**) correspond to the 5-days exposed plants. The lines represent the reported models’ regression lines and the dots are the data points. In (**A**,**B**) data points were vertically jittered to improve visualization. The regression line in (**C**) is not colour-coded by treatment because ozone treatment was not a predictor in the reported model. The p-values for each predictor in the reported models are shown.

The eggs took between 4 and 8 days to hatch (mean 5.8 days) and this was highly dependent on the temperature during the egg stage (Figs. 4A,B, Table 1), with higher temperatures reducing the necessary time to complete the egg stage (t = −17.27, p < 0.001 and t = −4.157, p < 0.001, for 1- and 5-day exposed plants respectively). Eggs laid on plants previously exposed to ozone took, on average, longer to hatch (t = 2.127, p = 0.040 and t = 2.017, p = 0.062, for 1- and 5-day exposed plants respectively).

Temperature during the caterpillars development was the variable that affected caterpillar weight the most (t = 22.950, p < 0.001 and t = 12.102, p < 0.001, for caterpillars reared on 1- and 5-day exposed plants, respectively). Nine days into the caterpillar stage, no effect of ozone was observed on the weight of caterpillars reared on plants exposed for 1 day (Fig. 4C). However, the caterpillars reared on plants exposed to ozone for 5 days were lighter than those reared on the corresponding control plants, but only when the temperature during the caterpillar stage was high (Fig. 4D; Treatment x temperature during the caterpillar stage, t = −2.856, p = 0.005).

## Discussion

Exposure of plants to high levels of ozone has been shown to alter the interactions between plants and insect herbivores. However, the duration of the exposure and interactions with ambient temperature have not yet been considered as factors affecting plant-herbivore interactions. In this study, longer exposures to ozone affected the quality of *Sinapis arvensis* plants more strongly, with subsequent stronger effects on the interactions with the herbivore *Pieris brassicae. P. brassicae* butterflies avoided ozone exposed plants for oviposition. Despite a positive effect of ozone exposure on the survival of the eggs, the number of hatched caterpillars was lower on ozone-exposed plants and the caterpillars performed less well when feeding on them, particularly at higher ambient temperatures, a climate scenario that is likely to become more common in the future.

Increasing the duration of exposure of *S. arvensis* plants to ozone resulted in avoidance of these plants by *P. brassicae* butterflies when choosing oviposition sites. This is likely due to alterations in the chemical cues produced by the plant, particularly changes in the chemicals of the leaf boundary layer, that are often used in food plant acceptance^30^. Our study is in accordance with some other studies addressing the plant-mediated effects of ozone on the oviposition preference of insect herbivores that show that the insects prefer to lay eggs on control plants^6,36^. In other studies, exposure of the plant to ozone had no effect on oviposition preference^11,35^, but, as we observed in the present study, this could be a matter of duration of exposure. In studies where the plants were presented to the insects during the exposure, some insects had a preference for laying eggs on control plants^37^ whilst others preferred ozone-exposed plants^35^.

Plants may react to egg deposition by a hypersensitive response^32^. In our study, the survival of the eggs was positively affected by ozone. We hypothesize that the exposure to an abiotic stress (ozone) prior to egg deposition may have inhibited a defence response from the plant in ozone-exposed plants, leading to a positive effect of ozone in the egg survival rate. Griese *et al*.^53^ showed that the expression or severity of the hypersensitive response does not increase with an increased number of eggs laid, but single-laid eggs are more susceptible to it than eggs laid in clusters and eggs laid in smaller clusters have a tendency to be more susceptible than eggs laid in bigger clusters. This is presumably because they are more vulnerable to desiccation. Although we did not specifically register survival per cluster, there was a positive correlation between the number of eggs per plant and the average number of eggs per cluster (r = 0.75). We consider that an increased susceptibility of eggs in smaller clusters to a defence response may be the reason why, in this study, the egg survival rate was higher on plants with more eggs laid on them. Despite the positive effect of ozone on egg survival, the number of caterpillars per plant after hatching was still higher for control plants than for ozone-exposed plants, showing an overall negative effect of ozone.

The effect of ozone on caterpillar performance was also negative, but only for longer exposures and only when the caterpillars were reared at higher ambient temperatures. This gives rise to two non mutually exclusive hypothesis: (1) higher temperatures intensify the response of the plants to ozone with consequences to their nutritional value and/or to their level of toxicity, therefore affecting the caterpillars development or (2) higher temperatures increase the metabolic rate of the caterpillars, leading caterpillars that have similar weights at hatching to diverge faster, and therefore to show an indirect response to ozone. In any of these cases, the lower weight of caterpillars reared on plants exposed to ozone for 5 days may mean that the caterpillars are growing into lighter, weaker pupae or that the caterpillars will take longer to pupate, and therefore that their life cycle will be extended. The latter was the case in a study by Jondrup *et al*.^39^, where they observed that caterpillars reared on ozone-sensitive plants exposed to ozone reached the same final weight, but took longer to pupate than the caterpillars reared on the control plants. On the other hand, Couture *et al*.^10^ observed that caterpillars showed decreased growth when fed foliage from trees growing under elevated ozone conditions. In Khaling *et al*.^5^ both phenomena occurred: caterpillars reared on ozone-exposed plants took longer to pupate and the pupae were lighter. If the reduced weight of the caterpillars reared on ozone-exposed plants shown here, translate into a longer caterpillar stage, together with the fact that the egg stage was also longer for eggs laid on ozone-exposed plants, the herbivores will have longer life cycles. Consequently, the number of generations produced per year may decrease and the predation or parasitism risk during the developmental stage may increase. Plant-mediated effects of ozone on caterpillar performance are not globally negative: Bolsinger *et al*.^38^ showed a higher relative growth rate of caterpillars when reared on plants exposed to ozone and Jackson *et al*.^27^ observed a tendency for increased growth of caterpillars fed with plants grown under elevated ozone conditions. Kopper *et al*.^54^ observed that ozone had no effect on the performance of caterpillars reared on trees growing under elevated ozone conditions and Jondrup *et al*.^39^ also saw no effect of ozone on caterpillars reared on resistant and wild type lines. When coupled with information about the nutritional state of the host, some studies suggest that alterations in caterpillar weight are related to changes in the nitrogen content of the host’s leaves, whether the effect of ozone was negative^5,9,10,12^ or positive^27^. Interestingly, in no-choice tests, caterpillars either consumed similar amounts of plant material irrespective of plant treatment^39^ or consumed more ozone-exposed plant material than control plant material^14,36^, which could indicate a mechanism to compensate for the reduction in nutritional value. However, in dual-choice feeding tests, herbivores also consumed more ozone-exposed plant material^3,5^ suggesting that changes in palatability may be the reason for the modified consumption.

In this study, the butterflies laid more eggs on control plants than on ozone-exposed plants, the same plants that later led to a better caterpillar performance. This is in agreement with the preference-performance hypothesis^55^ which states that females choose oviposition sites that maximize the fitness of their offspring. By doing so, and having fairly mobile adults, *P. brassicae* may be able to escape the detrimental effects of ozone on its development as long as small scale variability in ozone damage exists. On the other hand, not being able to move, plants cannot escape ozone. They suffer stress from both ozone exposure and herbivory. We did not test for feeding preferences, but if Khaling *et al*.^5^’s results on the increased consumption of ozone-exposed plant material would be applied in this situation, the fact that, as we observed, ozone-exposed plants had fewer caterpillars on them after hatching, may not be enough to compensate for the increased consumption. In our case ozone exposure seems to be a bad deal for both the plant and the herbivore. But even if one of them would be favoured by the exposure, the alterations in plant-herbivore interactions may affect the organisation of food webs, disturbing the balance of ecosystems.

Overall, the direction and strength of the herbivore response to ozone-exposed plants seem to vary between plant-insect systems. This variability may be caused by (1) different sensitivity to ozone between plant species, varieties or growth stages, (2) different susceptibility of the herbivores to the changes that ozone triggers in the plant or (3) different ozone exposure levels tested. The degree of sensitivity of a plant to ozone determines the exposure level that causes measurable changes in the plant which modify the plant’s interaction with its herbivores. In this study, both the plant and the herbivore were sensitive enough so that ozone effects could be observed on the herbivore life cycle at the ozone levels studied. Our results also suggest that the effects of ozone on plant-insect interactions are cumulative, since ozone affected oviposition and caterpillar performance when the plants were exposed for 5 days but not when plants were exposed for 1 day. However, Agathokleous *et al*.^56^ proposed that a plant does not respond linearly to ozone. A plant’s response to ozone could also follow a hormetic model, with low doses being beneficial to plants and detrimental effects only being observed when the ozone dose exceeds the NOAEL (no-observed-adverse-effects level). In the present study, the detrimental effects on plant-insect interactions observed for an exposure of 120 ppb ozone, 6 h/day for 5 days reveals that this level of ozone is beyond the NOAEL for this plant-herbivore system.

Our results identify that relatively low concentrations of ozone affect plant-herbivore interactions. AOT40 (Accumulated Ozone exposure over a Threshold of 40 ppb) is an index defined by the European Union (EU) for the protection of the vegetation. It is determined by calculating the sum of the difference between hourly concentrations greater than 40 ppb and 40 ppb over a given period using hourly values measured between 8 h 00 and 20 h 00 CET. In the Directive on Ambient Air Quality^57^, the EU pointed to 6000 µg/m3.h (~3000 ppb.h) as the long-term objective to be reached. In our 5-day-long treatments, the calculated AOT40 is ~2300 ppb.h, a level well below EU’s objective. However, as our results show, this level of exposure was already enough to cause damaging effects on the plants (visible injury) as well as to affect plant-herbivore interactions (oviposition and caterpillar performance). This points to the need of reviewing the European legislation on air quality, because currently it does not account for the damaging effect of acute ozone exposure, that seems to be important at least for annual plants like the one we used. Importantly, our data indicate that more frequent ozone peaks combined with higher temperatures, as predicted for a future with ongoing global warming and environmental pollution, will reinforce the negative effects of ozone on plant-herbivore interactions.

In summary, we showed that exposing *S. arvensis* to ozone affects several parameters of the life cycle of its herbivore *P. brassicae*. Our results reveal that a more severe exposure to ozone, especially when combined with higher temperatures, strengthens the effects of the pollutant on plant-herbivore interactions. Because plants vary in their sensitivity to ozone and herbivores vary in their susceptibility to changes in the plants, the alterations in plant-herbivore interations may vary in strength and direction between plant-herbivore systems, affecting the organisation of food webs and possibly disturbing the balance of ecosystems. This accentuates the need to implement measures to reduce the emission of precusors that could lead to ozone peaks such as the ones tested here, particularly in parts of the world where the use of fossil fuels is still increasing.

## Supplementary information

Supplementary Material

## Data Availability

The data generated during the current study are available from the authors on reasonable request.
