# Supplementary material for: Plant-mediated effects of ozone on herbivores depend on exposure duration and temperature
Source: Sci Rep. 2019 Dec 27;9:19891. doi: 10.1038/s41598-019-56234-z (PMC6934497; doi:10.1038/s41598-019-56234-z)
Supplement: Supplementary file 1 — Supplementary Material [file 41598_2019_56234_MOESM1_ESM.docx]

Plant-mediated effects of ozone on herbivores depend on exposure duration and temperature

Laura Duque^1*^, Erik H. Poelman^2^, Ingolf Steffan-Dewenter^1^

Department of Animal Ecology and Tropical Biology, Biocenter, University of Würzburg, Würzburg, Germany

2 Laboratory of Entomology, Wageningen University, Wageningen, The Netherlands

* Correspondence and requests for materials should be addressed to L.D. (laura.duque@uni-wuerzburg.de or lauraduque.science@gmail.com)

Table S1 – Ozone concentration (mean ± standard deviation) and accumulated exposure to ozone (AOT40) in the ozone chamber between 11h00 and 17h00, during the treatment days of each round.

| Round - Duration of treatment | [O3] (ppb) | AOT40 (ppb.h) |
| --- | --- | --- |
| 1 - 1 day | 112 ± 14 | 437 |
| 2 - 1 day | 113 ± 11 | 437 |
| 3 - 1 day | 119 ± 9 | 472 |
| 4 - 1 day | 119 ± 10 | 473 |
| 5 - 1 day | 122 ± 10 | 494 |
| 6 - 1 day | 120 ± 11 | 479 |
| 1 - 5 days | 117 ± 8 | 2298 |
| 2 - 5 days | 116 ± 9 | 2274 |
| 3 - 5 days | 116 ± 9 | 2267 |
| 4 - 5 days | 114 ± 10 | 2209 |
| 5 - 5 days | 114 ± 9 | 2230 |
| 6 - 5 days | 114 ± 9 | 2231 |
| 7 - 5 days | 117 ± 9 | 2312 |

Table S2. Summary of the models with ΔAICc < 2 for the number of eggs laid per plant in dual-choice assays

|  |  |  |  | Predictors | | | | | | | |
| --- | --- | --- | --- | --- | --- | --- | --- | --- | --- | --- | --- |
| Oviposition preference | Model | ΔAICc | weight | Treatment | | Fungal Infection | | Plant height | | Treat * Fungal Inf | |
|  |  |  |  | z | P | z | P | z | P | z | P |
| 1 day | Model 1 | 0.00 | 0.301 | ---------- | ---------- | -2.346 | 0.019 | ---------- | ---------- | ---------- | ---------- |
|  | Model 2 | 0.17 | 0.276 | ---------- | ---------- | -2.713 | 0.007 | 1.447 | 0.148 | ---------- | ---------- |
| 5 days | Model 1 | 0.00 | 0.241 | -1.830 | 0.067 | -2.088 | 0.037 | ---------- | ---------- | ---------- | ---------- |
|  | Model 2 | 1.05 | 0.142 | ---------- | ---------- | -1.880 | 0.060 | ---------- | ---------- | ---------- | ---------- |
|  | Model 3 | 1.15 | 0.135 | -1.665 | 0.100 | ---------- | ---------- | ---------- | ---------- | ---------- | ---------- |
|  | Model 4 | 1.65 | 0.105 | ---------- | ---------- | ---------- | ---------- | ---------- | ---------- | ---------- | ---------- |

Table S3. Summary of the models with ΔAICc < 2 for the number of caterpillars on the plants

|  |  |  |  | Predictors | | | | | |
| --- | --- | --- | --- | --- | --- | --- | --- | --- | --- |
| Caterpillars on plants | Model | ΔAICc | weight | Treatment | | Fungal Infection | | Treat * Fungal Inf | |
|  |  |  |  | z | P | z | P | z | P |
| 1 day | Model 1 | 0.00 | 0.515 | ---------- | ---------- | -2.248 | 0.025 | ---------- | ---------- |
|  | Model 2 | 1.86 | 0.203 | ---------- | ---------- | ---------- | ---------- | ---------- | ---------- |
| 5 days | Model 1 | 0.00 | 0.499 | -2.209 | 0.027 | ---------- | ---------- | ---------- | ---------- |
|  | Model 2 | 1.68 | 0.215 | -2.242 | 0.025 | -0.832 | 0.405 | ---------- | ---------- |

Table S4. Summary of the models with ΔAICc < 2 for the duration of the egg stage

|  |  |  |  | Predictors | | | | | | | | | |
| --- | --- | --- | --- | --- | --- | --- | --- | --- | --- | --- | --- | --- | --- |
| Duration of the egg stage | Model | ΔAICc | weight | Treatment | | Temperature | | Number of eggs | | Treat * Temp | | Treat * Nr of eggs | |
|  |  |  |  | t | P | t | P | t | P | t | P | t | P |
| 1 day | Model 1 | 0.00 | 0.361 | 2.127 | 0.040 | -17.269 | < 0.001 | ---------- | ---------- | ---------- | ---------- | ---------- | ---------- |
|  | Model 2 | 0.81 | 0.240 | 1.496 | 0.143 | -12.650 | < 0.001 | ---------- | ---------- | -1.333 | 0.190 | ---------- | ---------- |
| 5 days | Model 1 | 0.00 | 0.385 | 2.107 | 0.062 | -4.157 | < 0.001 | ---------- | ---------- | ---------- | ---------- | ---------- | ---------- |
|  | Model 2 | 1.80 | 0.157 | 1.781 | 0.112 | -4.151 | < 0.001 | -0.817 | 0.430 | ---------- | ---------- | ---------- | ---------- |

Table S5. Summary of the models with ΔAICc < 2 for the egg survival rate

|  |  |  |  | Predictors | | | | | | | | | |
| --- | --- | --- | --- | --- | --- | --- | --- | --- | --- | --- | --- | --- | --- |
| Egg survival rate | Model | ΔAICc | weight | Treatment | | Temperature | | Number of eggs | | Treat * Temp | | Treat * Nr of eggs | |
|  |  |  |  | z | P | z | P | z | P | z | P | z | P |
| 1 day | Model 1 | 0.00 | 0.275 | 2.962 | 0.003 | 1.923 | 0.055 | 1.885 | 0.059 | -2.796 | 0.005 | ---------- | ---------- |
|  | Model 2 | 0.24 | 0.244 | -1.636 | 0.102 | ---------- | ---------- | -1.057 | 0.291 | ---------- | ---------- | 2.778 | 0.005 |
|  | Model 3 | 0.4 | 0.226 | 3.876 | < 0.001 | 1.958 | 0.050 | -3.609 | < 0.001 | ---------- | ---------- | ---------- | ---------- |
|  | Model 4 | 1.7 | 0.117 | -1.592 | 0.111 | 1.135 | 0.256 | -0.966 | 0.334 | ---------- | ---------- | 2.724 | 0.006 |
|  | Model 5 | 1.92 | 0.105 | 1.363 | 0.173 | 1.655 | 0.098 | -0.070 | 0.944 | -1.557 | 0.119 | 0.984 | 0.325 |
| 5 days | Model 1 | 0 | 0.359 | 2.352 | 0.019 | ---------- | ---------- | 2.633 | 0.008 | ---------- | ---------- | ---------- | ---------- |
|  | Model 2 | 1.42 | 0.176 | 2.316 | 0.021 | -1.037 | 0.300 | 2.633 | 0.008 | ---------- | ---------- | ---------- | ---------- |
|  | Model 3 | 1.69 | 0.154 | 1.611 | 0.107 | ---------- | ---------- | 1.892 | 0.059 | ---------- | ---------- | -0.886 | 0.375 |

Table S6. Summary of the models with ΔAICc < 2 for the caterpillar weight

|  |  |  |  | Predictors | | | | | | | | | | | | | |
| --- | --- | --- | --- | --- | --- | --- | --- | --- | --- | --- | --- | --- | --- | --- | --- | --- | --- |
| Larval weight | Model | ΔAICc | weight | Treatment | | Temperature | | Number of eggs | | Fungal infection | | Treat * Temp | | Treat * Nr of eggs | | Treat * Fung Inf | |
|  |  |  |  | t | P | t | P | t | P | t | P | t | P | t | P | t | P |
| 1 day | Model 1 | 0.00 | 0.267 | ---------- | ---------- | 22.950 | < 0.001 | ---------- | ---------- | ---------- | ---------- | ---------- | ---------- | ---------- | ---------- | ---------- | ---------- |
|  | Model 2 | 1.49 | 0.127 | ---------- | ---------- | 7.327 | < 0.001 | ---------- | ---------- | -0.720 | 0.474 | ---------- | ---------- | ---------- | ---------- | ---------- | ---------- |
|  | Model 3 | 1.73 | 0.112 | -0.532 | 0.596 | 22.829 | < 0.001 | ---------- | ---------- | ---------- | ---------- | ---------- | ---------- | ---------- | ---------- | ---------- | ---------- |
|  | Model 4 | 1.95 | 0.101 | ---------- | ---------- | 22.194 | < 0.001 | -0.277 | 0.783 | ---------- | ---------- | ---------- | ---------- | ---------- | ---------- | ---------- | ---------- |
| 5 days | Model 1 | 0.00 | 0.418 | 2.760 | 0.007 | 12.102 | < 0.001 | ---------- | ---------- | -3.141 | 0.002 | -2.856 | 0.005 | ---------- | ---------- | ---------- | ---------- |
|  | Model 2 | 1.50 | 0.198 | 2.839 | 0.005 | 11.934 | < 0.001 | ---------- | ---------- | -3.005 | 0.003 | -2.926 | 0.004 | ---------- | ---------- | 0.714 | 0.477 |

Table S7. Number of replicates for each research question.

| Duration of the treatment | 1 day | | 5 days | |
| --- | --- | --- | --- | --- |
| Treatment | Ct | O_3_ | Ct | O_3_ |
| Plant injury | 48 | 48 | 56 | 56 |
| Number of laid eggs | 35 | 35 | 45 | 45 |
| Duration of the egg stage | 22 | 20 | 27 | 21 |
| Egg survival rate | 23 | 20 | 28 | 21 |
| Number of caterpillars per plant | 35 | 35 | 45 | 45 |
| Caterpillar weight | 330 | 374 | 531 | 468 |

Ct-control treatment


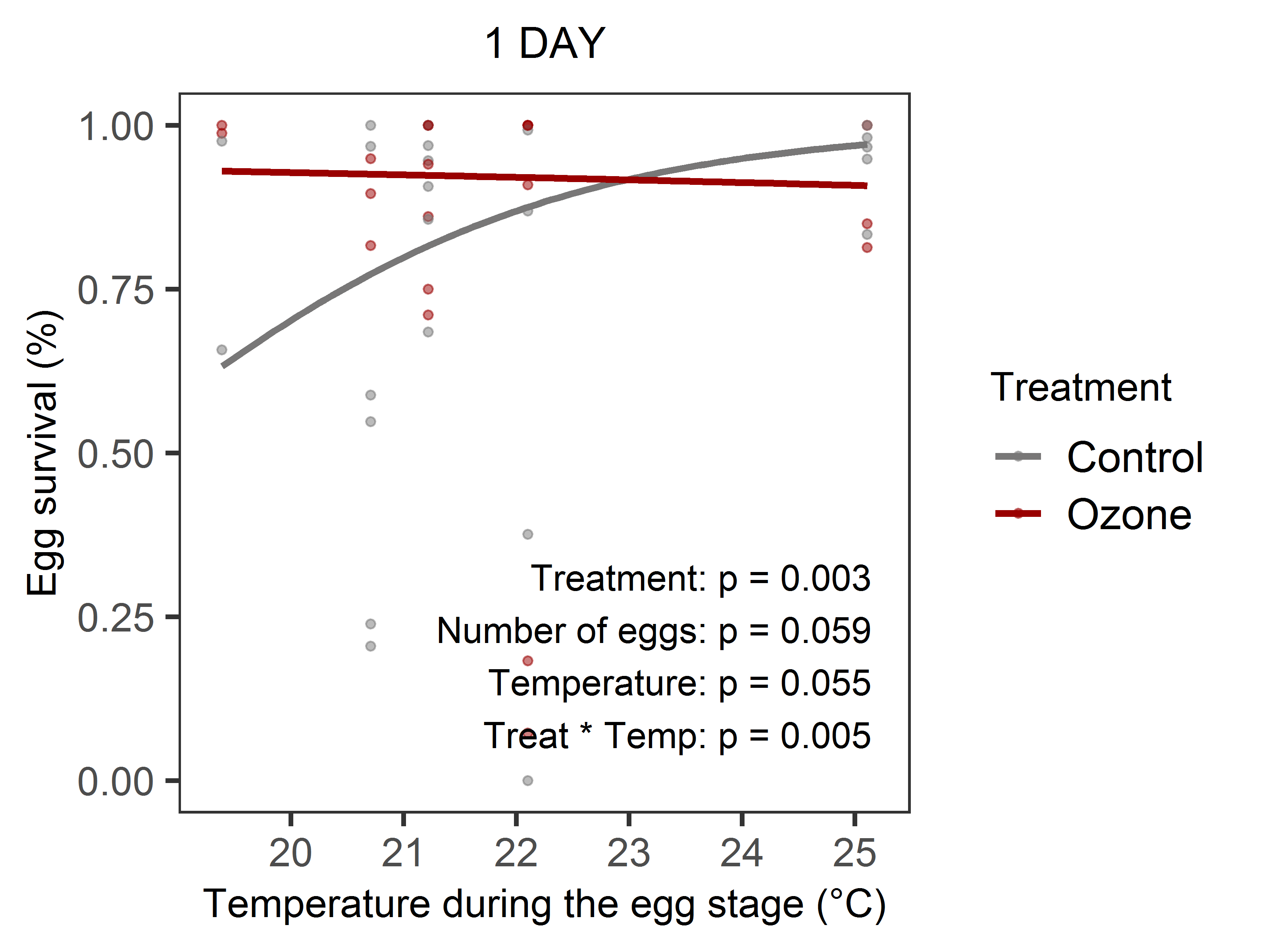


Figure S1 - The effect of ozone and temperature on the survival rate of eggs laid on 1-day treated plants. The lines represent the reported model’s regression lines and the dots are the data points. The p-values for each predictor in the reported model are shown.
